# Electrocardiographic wave variation among adults in Malaysia

**DOI:** 10.1186/1471-2458-14-S1-O7

**Published:** 2014-01-29

**Authors:** Mohd Hasni Jaafar, Jamal Hisham Hashim, Noor Hassim Ismail

**Affiliations:** 1 Department of Community Health, University Kebangsaan Malaysia Medical Centre, Jalan Yaakub Latif, Bandar Tun Razak, 56000 Cheras, Kuala Lumpur, Malaysia; 2 United Nations University International Institute for Global Health, 56000 Cheras, Kuala Lumpur, Malaysia (UNU-IIGH

## Background

Cardiovascular disease is among the main causes of morbidity and mortality in adult population in Malaysia. Since 10 years ago, it became the most important reason for hospitalisation in government hospitals. Non-invasive 12-lead resting electrocardiograph (ECG) has been recognised internationally as a simple, yet reliable surveillance tool to detect any cardiomyopathy condition. The objective of the study was to determine the varieties of ECG waves among healthy adult population in this country.

## Materials and methods

This cross-sectional study was conducted in a number of rural areas in Selangor. A total of 393 respondents agreed verbally to participate in the study and for their ECG to be recorded. A total of 255 women (65%) and 138 men (35%) were involved in this study which started in January 2012 until June 2012. The mean age for females was 51 years old while the mean age for males was 56 years old, with no significant gender difference (p > 0.05).

## Results

A total of 192 (49%) respondents had at least one abnormal parameter in their ECG. Fourteen (3.6%) of them had prolonged QRS wave. The majority of them were males (64%) with a mean QRS complex of 130 (± 6.86) ms, compared to female (36%) who had longer mean QRS complex of 146 (± 40.19) ms. However, this gender difference was insignificant (t = 1.19, p = 0.25). A total of 128 (33%) respondents had prolonged QTc interval of more than 440 ms. Most of them were female respondents (n = 94, 89%) compared to males. A total of 107 (27%) respondents had abnormal T wave axis with the majority occurring among women (n = 74, 70%).

## Conclusions

ECG is a very useful and convenient tool for cardiovascular screening among adult population. It is sensitive enough to detect abnormal varieties of ECG waves, which may contribute to future morbidity and mortality rate of cardiovascular problems. The findings suggest further evaluation of ECG screening among general population in view of reducing burden of non-communicable diseases and hospitalisation.

